# Blood group determinates incidence for pancreatic cancer in Germany

**DOI:** 10.3389/fphys.2013.00118

**Published:** 2013-05-24

**Authors:** U. Pelzer, F. Klein, M. Bahra, M. Sinn, B. Dörken, P. Neuhaus, O. Meyer, H. Riess

**Affiliations:** ^1^Department of Hematology/Oncology, Comprehensive Cancer Center, Charité – Universitätsmedizin BerlinBerlin, Germany; ^2^Department of Surgery, Charité – Universitätsmedizin BerlinBerlin, Germany; ^3^Institute for Transfusion Medicine, Charité – Universitätsmedizin BerlinBerlin, Germany

**Keywords:** pancreatic cancer, risk factor, ABO blood-group system, determination, genome

## Abstract

**Background:** Genetic risk factors for sporadic pancreatic cancer are largely unknown but actually under high exposure. Findings of correlations between the AB0 blood group system (Chromosome 9q34,1—q34,2) and the risk of pancreatic cancer (PC) in patients from Asia, America and south Europe have already been published. So far it is unclear, whether this correlation between blood group an PC incidence can be found in German patients as well.

**Methods:** One hundred and sixty-six patients who underwent a resection of PC were evaluated in a period between 2000 and 2010. Blood group reference distribution for the German population is given as: 0: 41%; A: 43%; B: 11%; AB: 5%; Rhesus positive: 85%; Rhesus negative: 15%. Analyses were done using the non-parametric Chi^2^-test (*p*-value two sided; SPSS 19.0).

**Results:** Median age was 62 (34–82) years. Gender: female 73/44%; male: 93/56%. Observed blood group proportions: 0: 43 (25.9%)/A: 94 (56.6%)/B: 16 (9.6%)/AB: 13 (7.8%)/Rhesus positive: 131 (78.9%)/negative: 35 (21.1%). We detected a significant difference to the German reference distribution of the AB0 system (Chi^2^ 19.34, df 3, *p* < 0.001). Rhesus factor has no impact on AB0-distribution (Chi^2^ 4.13, df 3, *p* = 0.25), but differs significantly from reference distribution—probably due to initial AB0-variation (Chi^2^ 4.82, df 1, *p* = 0.028). The odds ratio for blood group A is 2.01 and for blood group 0 is 0.5.

**Conclusions:** The incidence of PC in the German cohort is highly associated with the AB0-system as well. More patients with blood group A suffer from PC (*p* < 0.001) whereas blood group 0 was less frequent in patients with PC (*p* < 0.001). Thus, our findings support the results from other non-German surveys. The causal trigger points of this carcinogenesis correlation are still not known.

## Introduction

Advanced pancreatic cancer holds one of the highest mortality rates of any cancer, with corresponding 5 year survival rate of less than 5% (Adler et al., 2007; Pelzer, 2008). It remains one of the leading causes of cancer-related deaths worldwide, reflected by an incidence of 277,668 new cases and almost the same mortality rate (266,029 cases) per year (Jemal et al., 2010). Due to early disease symptoms being absent, only up to 20% of patients can have their cancer resected with curative intent, however, probably due to early lymphogenic spread or micro metastasis, the 5-year overall survival rate of resected patients is only 15–22% in spite of adjuvant treatment. An effective screening method or test for this devastating cancer is still missing. Established risk factors include a family history of pancreatic cancer, a medical history of hereditary pancreatitis, diabetes type II and cigarette smoking. Established research groups seeking for predefined genome aberrations correlated to pancreatic cancer (Amundadottir et al., 2009; He et al., 2013). In recent time several studies investigating the possible correlation of the AB0 blood group system to pancreatic cancer (Yeo and Lowenfels, 2012) were published. Correlations were found in many populations, exemplary in Turkish patients (Engin et al., 2012), Korean patients (Woo et al., 2013), Japanese patients (Nakao et al., 2011), Italian patients (Iodice et al., 2010), and North American patients (Greer et al., 2010; Wolpin et al., 2010). But there is no overall accordance in all populations. For instance in Chinese patients publications showed inconsistent results without detection of correlations on the one hand (Gong et al., 2012) and proof of coherence on the other hand (Ben et al., 2011). These assured correlations are not consistent over all malignancies (Khalili et al., 2011). Currently no published survey data of German patients or a central Europe cohort exists which could help clarify the possible coherence. For further detection of causality it is important to know whether these findings are valid for German patients as well, therefore we conducted this investigation.

## Materials and methods

Patients who underwent a resection of PC were evaluated in a period between 2000 and 2010. All patients suffered from histologically confirmed pancreatic cancer. Blood type assay from 166 patients (AB0 antigen and Rhesus antigen) were conducted. As reference cohort, healthy blood donors from our department of transfusion medicine were tested, whose blood types showed the same distribution as the reference distribution of the German population. Reference distribution was given as: blood group 0 41%; blood group A 43%; blood group B 11%; blood group AB 5%; Rhesus antigen positive 85% and Rhesus antigen negative 15%. In addition to descriptive analyses non-parametric Chi^2^-tests (*p*-value two sided; SPSS 19.0) were used for comparisons.

## Results

**Figure 1:**
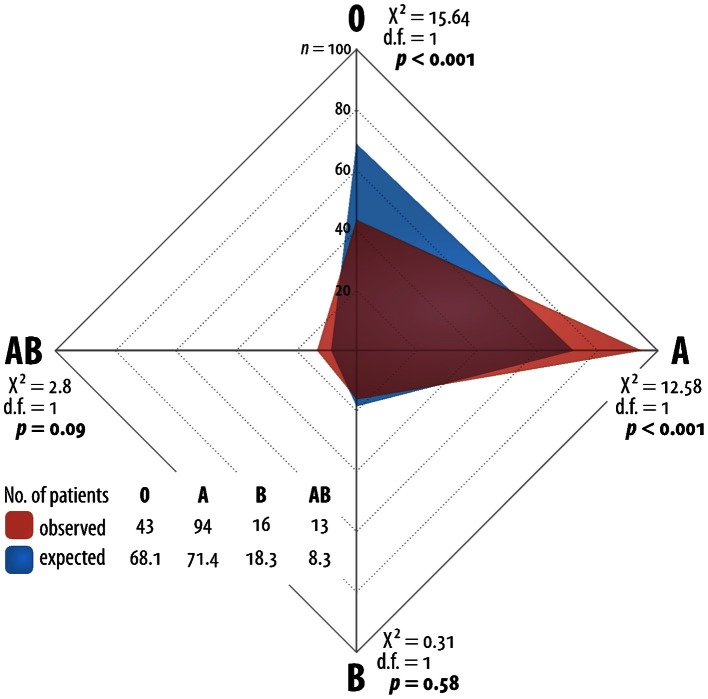
**Network grafic—statistical distribution of blood group in Germany and for German patients with pancreatic cancer**.

The present, non-selected population of patients with pancreatic cancer reflects the general population with PC in Germany as you can find in many trials. The median age was 62 (34–82) years. The gender distribution favors male patients with a percentage of 56% male patients to 44% female patients (Table 1). We observed blood group 0 in 43 (25.9%) patients, blood group A in 94 (56.6%) patients, blood group B in 16 (9.6%) patients and blood group AB in 13 (7.8%) patients. These observations differ significantly from the reference distribution of the AB0 system (Chi^2^ 19.34, df 3, *p* < 0.001). The absolute differences to the expected AB0-distribution were minus 25 patients for blood group 0, plus 23 for blood group A, minus two patients for blood group B and plus five patients for blood group AB. The odds ratio for blood group A is 2.01 and for blood group 0 is 0.5. The Chi^2^-tests for the single AB0-characters were as follow: for 0 (Chi^2^ 15.64, df 1, p < 0.001), for A (Chi^2^ 12.58, df 1, *p* < 0.001), for B (Chi^2^ 0.31, df 1, *p* = 0.58), and for AB (Chi^2^ 2.80, df 1, *p* = 0.09) (Figure 1, Table 2).

**Table 1:** **Patients' characteristics**.

**Characteristic**	**Patients**
Included	166
Age: median [range]	62 [34–82] years
<60 years	63
60–70 years	69
>70 years	34
Gender	
Female	73
Male	93
Biopsy proven adeno-carcinoma (pancreas)	166

**Table 2:** **Calculations between single characters of group antigens**.

	**Observed patients**	**Expected patients**	
0	43	68.1	Chi^2^ 19.34 df 3
			*p* < 0.001
A	94	71.4	
B	16	18.3	
AB	13	8.3	
Rh. neg.	35	24.9	Chi^2^ 4.82 df 1
			*p* < 0.028
Rh. pos.	131	141.1	
Overall	166	166	

Furthermore we observed the positive rhesus antigen in 131 (78.9%) patients and the negative rhesus antigen in 35 (21.1%) patients. The Rhesus factor has no significant impact on the AB0-distribution (Chi^2^ 4.13, df 3, *p* = 0.25) within the observed cohort. As compared to the reference cohort, the distribution of the Rhesus factor resulted in a significant difference (Chi^2^ 4.82, df 1, *p* = 0.028). This observation is possibly boosted due to the initial AB0-variation (Table 3).

**Table 3:** **Calculations of AB0/Rhesus independence**.

**Blood group antigen**	**Rhesus antigen**		**All**
	**Negative**	**Positive**	
**0**			
obs. pts.	5	38	43
exp. pts.	9.1	33.9	
**A**			
obs. pts.	21	73	94
exp. pts.	19.8	74.2	
**B**			
obs. pts.	5	11	16
exp. pts.	3.4	12.6	
**AB**			
obs. pts.	4	9	13
exp. pts.	2.7	10.3	
**All**			
obs. pts.	35	131	166
exp. pts.			

Rhesus/AB0-proportions are independent (Chi^2^ 4.13, df 3, p = 0.248).

## Discussion

In spite of recent advantages in the treatment modalities, likewise the FOLFIRINOX 1st-line regimen (Conroy et al., 2011), the OFF 2nd-line treatment (Pelzer et al., 2011) or the latest data from the gemcitabine/nab-paclitaxel 1st-line treatment, patients suffering from pancreatic adenocarcinoma still have the poorest survival outcome among cancer illnesses at all. Because of the heavy difficulties in the treatment of advanced disease, increased effort was dedicated to detect risk factors and causalities in the carcinogenesis to diagnose patients at earliest point of disease. Descriptions of observed correlations form the basis for further investigations.

The characteristics of our observed patients were in accordance with the appearance of the German clinician in terms of gender and age (Adler et al., 2007). Thus, this cohort is representative for patients with pancreatic carcinoma in Germany. Our findings are not completely identical with the observations of other research groups, but agree with risk lowering in patients with blood group 0. The Korean survey displayed an increased risk for the population with non-blood 0 character (Woo et al., 2013), Turkey findings showed higher risk of patients with blood group A and a lower risk of patients with blood group AB (Engin et al., 2012) whereas Chinese patients were interestingly investigated without blood group risk correlation (Gong et al., 2012). There are some attempts to explain the observed correlations, mainly based on the assumption of collocated signal cascade triggers. The chronic pancreatitis is known to be a risk factor for carcinogenesis. A misfit is that chronic pancreatitis was found to correlate with blood group 0 (Greer et al., 2011) which on the other hand lower the risk of pancreatic cancer. Another way of sourcing is the infection triggered chronic inflammation. Helicobacter pylori infection is also associated with the AB0 genotype mainly due to the AB0 antigen expressions on gastrointestinal epithelium and therefore better adhesion for the Helicobacter colonization. The positive association between the AB0 expression and duodenal and gastric ulcer as well as gastric cancers may base on effects of gastric and pancreatic secretory function disorders. This could have an additional impact on the carcinogenicity of dietary- and smoking-related N-nitrosamine exposures, and thus risk of pancreatic cancer (Risch, 2012).

Furthermore, venous thromboembolic events are also associated with pancreatic cancer known as trousseaus syndrome, first described in 1865. Is there a common base? Von Willebrand factor (vWF) is one mediator for this cause. It has the blood group antigens A and/or B on its surface and carries factor VIII and protects it from degradation. Blood group A1 and B educes a higher level of vWF and factor VIII. Thrombosis may appear as an early observed symptom of the subsequent diagnosis of pancreatic cancer. The activated coagulation was formerly described as an additional trigger associated with poor prognosis and increased angiogenesis. There is supposedly a combined cascade of carcinogenesis activation (Maisonneuve et al., 2009). But, maybe as a result of earlier detection or more effective therapy of thrombosis and thromboembolic events, recent studies showed no survival disadvantage for cancer patients suffering thrombosis (Riess et al., 2008; Agnelli et al., 2009).

It is noteworthy that other cancer types do not have stringent correlations to the AB0-antigen (Iodice et al., 2010), indicating it to be a special observation in pancreatic cancer disease. But of all above, the feasible research hypothesis is that the single base deletion that generates the 0 blood group underlies the association signal. Additional mapping and laboratory work is mandatory to determine which variants account for the observed correlation (Amundadottir et al., 2009).

The discovery of additional genetic risk factors for this highly lethal cancer type may contribute to novel risk stratifications and advances in prevention, early detection and therapeutic approaches to pancreatic cancer.

Based on these representative data, we plan a genome mapping project of available data from our adjuvant studies (CONKO 001/005/006), which is under recent approval of the German Society of Cancer.

## Conclusions

The incidence of pancreatic cancer in Germany is significantly associated with the AB0-blood group system. More patients with blood group A suffer from pancreatic cancer (*p* < 0.001) whereas blood group 0 was less frequently observed in patients with pancreatic cancer (*p* < 0.001). Genetic variations in the AB0 locus of 9q34 may influence the pancreatic carcinogenesis and increase the risk for patients with blood group A and tapering the risk for patients with blood group 0.

### Author contributors

U. Pelzer, M. Bahra, and H. Riess were responsible for the concept and design of the study and the writing of the manuscript. Collecting data and further analysis and interpretation was done by U. Pelzer, M. Bahra, F. Klein, M. Sinn, and H. Riess. O. Meyer, H. Riess, and B. Dörken provided staff and facilities for the investigation. All authors were involved in the provision of patients and the collection and collation of data. All authors reviewed the manuscript and gave their approval.

### Conflict of interest statement

The authors declare that the research was conducted in the absence of any commercial or financial relationships that could be construed as a potential conflict of interest.
